# Dual Regulation of Mitochondrial Complexes by H_2_S via *S*-Sulfhydration Controls Respiration in Type 1 Diabetic Hearts

**DOI:** 10.3390/biom15081197

**Published:** 2025-08-20

**Authors:** Tong Su, Li Han Zhu, Jun Xian Liu, Li Yuan Jin, Huixing Cui, Longhao Yu, Yin Hua Zhang

**Affiliations:** 1 Yanbian University College of Medicine, Yanbian University Hospital, Yanji 133000, China; sutong19941101@gmail.com (T.S.);; 2 Department of Physiology & Biomedical Sciences, Ischemic/Hypoxic Disease Institute, Seoul National University College of Medicine, Seoul 03080, Republic of Korea; 3 Department of Cardiology, Nanxishan Hospital of Guangxi Zhuang Autonomous Region, Guilin 541002, China

**Keywords:** hydrogen sulfide, mitochondria, *S*-sulfhydration, mitochondrial complexes, ATP synthase, type 1 diabetes mellitus, ischemic reperfusion

## Abstract

Hydrogen sulfide (H_2_S) has been established to regulate mitochondrial respiration and ATP production, but whether the regulation is through *S*-sulfhydration (-SSH) of mitochondrial complexes is not well understood. Recently, H_2_S is known to exert diverse and dose-dependent effects on mitochondrial complexes. However, the involvement of *S*-sulfhydration of each mitochondrial complex and the activities in diabetic hearts have not been revealed. Here, we conducted comprehensive investigations into *S*-sulfhydration and the activities of mitochondrial complexes I–V in normal and Streptozotocin (STZ)-induced type 1 diabetic (DM) heart mitochondria. Results showed that proteins of H_2_S-producing enzymes were downregulated in DM heart mitochondria, which was accompanied by reduced mitochondrial membrane potential (MMP), greater ROS, and lower complex I and V activities, reduced complex V-SSH in DM. In both groups, supplementation with the H_2_S donor NaHS increased the *S*-sulfhydration of all mitochondrial complexes, and the activities of complexes I–III and V were significantly increased but complex IV activity was reduced. Consequently, mitochondrial MMP, ROS, and ATP production were normalized with NaHS in DM, whereas inhibition of H_2_S generation increased mitochondrial ROS and reduced MMP via reducing complex activities in both groups. Ischemic reperfusion did not affect NaHS-increment of *S*-sulfhydration of complexes I–V, but significantly impaired complex V activity in DM. Collectively, H_2_S-dependent *S*-sulfhydration of mitochondrial complexes I–V in normal and DM heart mitochondria were involved in the activation of mitochondrial complexes I–III/V and the inhibition of complex IV, which control cardiac mitochondrial respiration and ATP production.

## 1. Introduction

Hydrogen sulfide (H_2_S) is an important signaling molecule that exerts critical physiological functions in various tissues including the heart. H_2_S is mainly synthesized from cysteine and homocysteine by three key enzymes: cystathionine γ-lyase (CSE or CTH), cystathionine β-synthase (CBS), and 3-mercaptopyruvate sulfurtransferase (3-MST or MPST) [1,2,3]. H_2_S has been implicated in maintaining cardiovascular homeostasis by modulating the functions of enzymes, receptors, and ion channels [4,5], predominantly through reversible modifications of cysteine *S*-sulfhydration, transferring sulfur molecule to the thiol (-SH) groups of target proteins into sulfhydration (-SSH) [5,6,7,8,9,10]. It is well acknowledged that *S*-sulfhydration is a prevalent post-translational modification, which has been associated with various catalytic responses of target proteins [7,8,11]. In metabolically active organs, such as in the liver, approximately 25–50% of proteins are subjected to *S*-sulfhydration [12]. Ca^2+^/calmodulin-dependent protein kinase II (CaMKII), a key regulator in heart failure and mitochondrial dysfunction, exhibits increased *S*-sulfhydration upon H_2_S supplementation, leading to its inhibition and the preservation of cardiac function [13]. Similarly, exogenous H_2_S enhances the *S*-sulfhydration of peroxisome proliferator-activated receptor-γ coactivator-related protein, upregulating peroxisome proliferator-activated receptor-γ coactivator-1, which promotes mitochondrial biogenesis and maintains cellular energy homeostasis [14]. Furthermore, *S*-sulfhydration has been shown to enhance the antioxidant capacity of glutathione in the nervous system, thereby providing protection from neuronal degeneration [15]. Additionally, exogenous H_2_S promotes ubiquitin-mediated degradation of SREBP1 to alleviate diabetic cardiomyopathy via *S*-sulfhydration of synoviolin1 [16]. Therefore, *S*-sulfhydration, as a post-transcriptional modification mechanism, plays essential roles in mediating the important physiological effects of H_2_S in biological systems.

Recent evidence strengthens the roles of H_2_S in the mitochondria, i.e., H_2_S maintains mitochondrial integrity and bioenergetics, as well as stabilizing mitochondrial function through reducing oxidative stress [17]. H_2_S serves as an electron donor for the mitochondrial electron transport chain via sulfide quinone oxidoreductase (SQOR) and downstream cytochrome c oxidase (Com-plex IV), thereby maintaining basal mitochondrial respiration [18,19,20,21]. Exogenous H_2_S donors have been shown to enhance Complex I activity [18,21], promote electron transport, and restore metabolic activities that are attenuated in metabolic diseases such as diabetes [22]. While Complex II may not directly interact with H_2_S, recent studies suggest that it participates in reverse electron transport through SQOR, leading to succinate accumulation [22,23,24]. However, H_2_S exhibits concentration-dependent biphasic effects on complex IV between 1-100 μM: low concentrations promote complex IV activity, while high concentrations inhibit oxygen utilization, limiting electron transport and ATP production [18,25]. Furthermore, H_2_S stimulates ATP synthase (Complex V) activity [26], thereby promoting biogenesis through *S*-sulfhydration-mediated regulation of Complex V [26].

Mechanisms of H_2_S regulation of cardiac mitochondrial complexes in DM are not clearly defined, although abundant evidence indicates reduced H_2_S levels in DM patients or in animal models [16,27,28]. It is possible that mitochondrial dysfunction in DM heart is manifested by reduced H_2_S and its regulations [29,30]. Therapeutic strategies with exogenous H_2_S donors showed promising effects, reducing endothelial cell dysfunction and oxidative stress [31], and as such, H_2_S is instrumental in maintaining coronary circulation and protection in DM hearts. Indeed, exogenous H_2_S supplementation has been shown to enhance mitochondrial complex activity and energy production [27]. Until recently, whether *S*-sulfhydration mediates H_2_S regulation of mitochondrial complexes and the activities of sham and type 1 DM hearts has remain under evaluation.

Here, we aimed to conduct a comprehensive study investigating H_2_S regulation of mitochondrial complexes in sham and type 1 DM rat hearts. The *S*-sulfhydration of mitochondrial complexes (I-V) and the modifications of complex activities under basal conditions and after ischemia-reperfusion were identified. This study clarifies that H_2_S producing enzymes were downregulated in DM rat hearts, which may be linked with reduced mitochondrial functions. A H_2_S donor, sodium hydrosulfide (NaHS), positively regulated *S*-sulfhydration of mitochondrial complexes I-V in sham and DM but elicited more pronounced responses in DM. NaHS increased the activities of complexes I–III and V and inhibited complex IV activity in both groups, maintaining mitochondrial respiration. Ischemia-reperfusion impaired complex V and mitochondrial respiration responses to H_2_S donor in DM. Therefore, dual regulations of mitochondrial complex activities by H_2_S is critical in controlling mitochondria-dependent oxidative stress and biogenesis in healthy and diabetic hearts.

## 2. Materials and Methods

### 2.1. Type 1 Diabetes Model

Animal experiments were conducted in accordance with the approved guidelines from the Institutional Animal Care and Use Committee of Seoul Medical University, South Korea [SNU-230413-3-2]. Sixty Sprague Dawley (SD, Koatech, Republic of Korea) rats were acclimated for three days and randomly divided into four groups: sham (*n* = 15), sham ischemic-reperfusion (*n* = 15), DM (*n* = 15), and DM ischemic-reperfusion (*n* = 15). Streptozotocin (STZ) was intraperitoneally injected at a dose of 55 mg/kg in a solution of 0.1 mol/L citric acid-sodium citrate buffer (pH 4.2–4.5) to induce DM [32]. The STZ was prepared on ice and was protected from light, and it was used within 30 min. Basic indicators such as food intake, water consumption, and urine output were tracked using metabolic cages, along with periodic measurements of body weight and blood glucose (Roche Accu-Chek Active Set meter). After 4 weeks, rats were anesthetized by aerosol, and heart tissues were collected.

### 2.2. Oral Glucose Tolerance Test (OGTT)

After measuring fasting blood glucose (FBG), a 1 ml syringe connected to a gastric needle was used to deliver glucose solution (2 g/kg) via standard intragastric procedures. Timing commenced upon completion of the administration, with blood glucose levels measured at 15, 30, 60, 90, and 120 min. The average of three measurements at each time point was calculated for data analysis.

### 2.3. Isolation of Mitochondria from Cardiac Tissue

Mitochondria were isolated from rat left ventricular tissue using a mitochondria isolation kit (Abcam, ab110168, UK). The heart tissue was minced, and 150 mg was mixed with 800 µL of isolation buffer in a pre-cooled glass homogenizer, ground for 30–40 strokes, and the tissue suspension was centrifuged at 1000× *g* for 10 min at 4 °C to collect the mitochondria. The pellet was discarded and the supernatant was centrifuged again at 12,000× *g* for 15 min at 4 °C, after which the supernatant was discarded. After discarding the supernatant, the mitochondrial pellet was washed twice with isolation buffer containing protease inhibitor cocktail and resuspended to a concentration of 1 µg/µL using the Bradford protein quantification method. Active mitochondria were isolated from fresh left ventricular tissue, followed by incubation with NaHS (100 μM) at 37 °C for 10–30 min according to specific experimental protocols, serving as H_2_S supplementation treatment for subsequent assays.

### 2.4. ROS Determination in the Mitochondria

ROS were measured using the Amplex™ Red Hydrogen Peroxide/Peroxidase Detection Kit (Invitrogen, A22188, Pittsburgh, PA, USA). Prepare the Amplex Red mix per well as follows: 0.5 µL of 10 mM Amplex Red storage solution, 2 µL of 10 U/mL HRP stock solution, 97.5 µL of 1× reaction buffer, 10 µL of 1 µg/µL mitochondrial sample, and 45 µL of configured substrate, filling to a total volume of 200 µL with MAS buffer (in mM: D-Mannitol, 220; Sucrose, 70; KH_2_PO_4_, 10; MgCl_2_, 5; HEPES, 2; EGTA, 1; 0.2% (*w*/*v*) BSA, pH 7.2 with 1M KOH). Adjust the volume of MAS buffer accordingly when additional reagents are added (such as H_2_S enzyme inhibitor: 400 µM DL-propargylglycine (PAG), 300 µM hydroxylamine (HA), 100 µM I3MT-3 (HMPSNE, Sigma, Ronkonkoma, NY, USA) (1). After mixing all components except for the substrate, incubate at 37 °C for 10 min. Subsequently, add the substrate, mix well, and transfer 200 µL of the sample to a 96-well plate. Set the fluorescence microplate reader to 37 °C with 10 min/30 sec cycles, Ex/Em = 540/580 nm. After the detection, add 1 µL of oligomycin (final concentration 25 μM) to each well to analyze ROS production rates in both basal and oligomycin-inhibited Complex V states.

### 2.5. Determination of MMP

TMRM (T5428, Sigma, USA) mix was prepared as follows: Combine 2 µL of 10 µM TMRM storage solution, 10 µL of 1 µg/µL mitochondrial sample, 45 µL substrate, and MAS buffer to a total of 200 µL. Adjust the volume of MAS buffer for any additional treatments while maintaining the total volume at 200 µL. Mix all components except the substrate and incubate at room temperature for 20 min. After incubation, add the substrate, mix well, and set the fluorescence reader to 28 °C with 10 min/30 sec cycles, Ex/Em = 540/580 nm. After each 10 min detection, 1 µL of oligomycin was added to each well to analyze MMP in both basal and oligomycin-inhibited Complex V states.

### 2.6. Determination of Mitochondrial Oxygen Consumption Rate (OCR)

Mitochondrial OCR was measured using Instech equipment (NeoFox, Ocean Optics, Dunedin, FL, USA) at 37 °C. Calibration was performed with DW and 0.5 mol/L NaS_2_O_4_ solution [33]. Experiment sample: Add 40 µL of 1 µg/µL mitochondria, 90 µL substrate, and MAS buffer to a total volume of 340 µL. Adjust MAS buffer volume for any additional treatments, maintaining the total volume to 340 µL. After adding all components, quickly close the lid and record. Calculations are based on oxygen consumption between 8–12%.

### 2.7. Determination of S-Sulfhydration Using Modified Biotin Switch Assay

A modified biotin-switch assay was carried out as described previously. Isolated mitochondria were lysed in Lysis HEN buffer (in mM: HEPES, 250; EDTA, 1; 1% Triton X-100; 0.1% SDS; 0.1% sodium deoxycholate) with freshly added neocuproine (0.1 mM) and 1X protease inhibitor. After homogenization and centrifugation (14,000 rpm, 5 min, 4 °C), the supernatant was used for BCA quantification. 500 μg of protein was blocked with MMTS blocking buffer (Sigma, USA) (dilute 9.439 µL of MMTS stock with 20 µL DMSO, then mix with 4.5 mL HEN buffer and 500 µL 25% SDS to prepare 5 mL of MMTS blocking buffer) and rotated at 50 °C/1400 rpm for 20 min. The protein was precipitated with cold acetone, washed twice with 70% acetone, and resuspended in HENS buffer SDS (25% *w*/*v*) 1 ml + HEN buffer 24 mL) containing 0.8 mM biotin-HPDP (to 200 μL biotin-HPDP, add 800 μL HENS buffer), adjusted to approximately 0.5–1 mg/mL protein. After 1 h of incubation in the dark, the protein was precipitated again with acetone and resuspended in 250 µL of HENS buffer and 750 µL of neutralization buffer (containing 20 mM HEPES, 100 mM NaCl, 1 mM EDTA, and 0.5% Triton X-100), leaving 15–30 µL for input. The sample was then added to streptavidin beads pre-washed with neutralization buffer and rotated overnight at 4 °C. The beads were then washed six to seven times with neutralization buffer containing high salt (20 mM HEPES, 600 mM NaCl, 1 mM EDTA, 0.5% Triton X-100). Subsequently, 35 μL of elution buffer (containing 1% β-mercaptoethanol, 100 mM NaCl, and 0.1% sodium deoxycholate) was added. The mixture was incubated at 37 °C and eluted by shaking at 1000 rpm for 30 min. After centrifugation, the supernatant was collected, 4× LDS sample buffer was added, and gel electrophoresis was performed.

### 2.8. Determination of Mitochondrial Complex Activity with ELISA Kits

The activities of mitochondrial complexes I-V were determined using enzyme-linked immunosorbent assay (ELISA) kits (Abcam, UK ab109721, ab109928, ab287844, ab109911, ab109714). The experiments were performed according to the manufacturer’ s instructions. The temperature and wavelength of the fluorescence microplate reader were pre-set to appropriate values. The plate was then read continuously, and the linear rate of absorbance change over time was calculated, as complex activity is proportional to the absorbance change. The reaction rate (mOD/min) was determined using the following formula: reaction speed (mOD/min) = (OD1 − OD2)/(T1 − T2).

### 2.9. Determination of Mitochondrial CBS Activity

CBS activity was measured from cardiac mitochondria using ELISA kits (MBS1604772). Reagents, standards, and samples were prepared at room temperature according to the manufacturer’s instructions. First, 50 μL of the standard solution was added to the standard wells, while 40 μL of samples was mixed with 10 μL of anti-CBS antibody and 50 μL of streptavidin-HRP in the sample wells. After a 60 min incubation at 37 °C, the plate was washed five times. Subsequently, 50 μL substrate solutions A and B were added, and the plate was incubated in the dark for 10 min. The reaction was halted by adding 50 μL of Stop Solution, and the optical density (OD) was measured at 450 nm.

### 2.10. H&E and TUNEL Staining

Tissue blocks were fixed in 4% formaldehyde, followed by dehydration, wax embedding, slicing, and baking. The slices were collected and stored for staining. They were dewaxed and rehydrated as needed. H&E staining: Deparaffinize and rehydrate sections. Stain in hematoxylin (5 min), then rinse. Differentiate in acid alcohol, then rinse. Blue in ammonia water. Stain in eosin (1 min), dehydrate, and mount.

TUNEL Assay Kit (Abcam, Cambridge, UK, ab66110): A 1 mL solution of 20 µg/mL Proteinase K was prepared by mixing 2 µL of Proteinase K (10 mg/mL), 998 µL of Tris-HCl (pH 8.0), and 50 mM EDTA. The sections were treated with 100 µL of this Proteinase K solution for 5 min, followed by a 5 min treatment with 4% formaldehyde in PBS. Next, a DNA labeling solution was prepared, consisting of 10 µL of TdT Reaction Buffer, 0.75 µL of TdT enzyme, 8 µL of Br-dUTP, and 32.25 µL of ddH2O. The slides were placed in a dark, moist chamber at 37 °C for 1 h for labeling. Afterward, the slides were rinsed and incubated with 100 µL of antibody solution (5 µL of Anti-BrdU-Red antibody and 95 µL of rinse buffer) in the dark at room temperature for 30 min. Following incubation, DAPI anti-fading mounting solution was added. The confocal microscope was set to excitation/emission wavelengths of 488/576 nm (for BrdU-Red) and 405/465 nm (for DAPI) for observation and imaging. The fluorescence intensity of apoptotic cells was analyzed using ImageJ software (Image j 1.8.0, National Institutes of Health, Bethesda, MD, USA).

### 2.11. Langendorff Perfusion and Mapping

SD rats were anesthetized with sodium pentobarbital (30 mg/kg, i.p.) and the hearts were removed and quickly mounted on a Langendorff perfusion system, and then perfused for 10 min at 37 °C with a nominal Ca^2+^-free solution (in mM: NaCl 135, KCl 5.4, MgCl_2_ 3.5, glucose 5, HEPES 5, Na_2_HPO_4_ 0.4, taurine 20, pH 7.4, NaOH). The perfusate was clamped for 30 min in the ischemic group, and perfusion was continued for 30 min in the non-ischemia group. After excision, the heart was perfused retrogradely with oxygenated Krebs–Henseleit solution at 37 °C via a Langendorff apparatus. Monophasic action potentials were recorded on isolated rat left ventricles using a 64-channel multi-electrode array (MEA) system (mapping lab) before and after the 30 min perfusion. Conduction velocity (CV) was calculated based on the spatial and temporal differences between electrode activation points. The absolute dispersion of activation was calculated to assess regional heterogeneity of conduction.

### 2.12. Western Blotting Detecting Protein Expression

Proteins were extracted from heart tissues or isolated mitochondria using lysis buffer (with phosphatase inhibitors, pH 7.4). Protein concentration was determined by BCA assay, and samples were prepared with 5 × loading buffer. Lysates were boiled at 95 °C for 10 min (or at 37 °C for total oxphos samples), separated by SDS-PAGE, and transferred to PVDF membranes. After blocking, membranes were incubated with primary antibodies overnight (antibody details are provided in Table A2: antibody list), followed by secondary antibodies (anti-mouse or anti-rabbit secondary antibodies). Blots were developed using ECL, and protein levels were quantified with Image J.

### 2.13. RNA Extraction and Quantitative Real-Time PCR

Total RNA was extracted from heart tissue using Qiazol Lysis Reagent (Catalog Number 79306, QIAGEN, Frankfurt, Germany). The concentration of purified RNA was determined with a NanoDrop spectrophotometer (Thermo Fisher, Pittsburgh, PA, USA). Primers for 18S RNA, PGC1-α, and other targets were synthesized by a commercial provider; primer details are provided in the table below (Table A2: Primer list).

Real-time PCR was performed using the Applied Biosystems 7500 system under the following conditions: SYBR Green (BIONEER 2× Green Star qPCR Master Mix) was used to amplify cDNA with the following parameters: 95 °C for 3 min, followed by 40 cycles of 95 °C for 10 s, 55 °C for 3 s, 95 °C for 10 s, and 65 °C for 5 s. Specificity was confirmed through melting curve analysis of the amplified products. Relative gene expression was calculated using the ΔΔCt method, with the formula 2^−ΔΔCt^.

### 2.14. Quantification and Statistical Analysis

Normality tests were performed on all data sets using the D’ Agostino and Pearson method in Prism GraphPad 8.0. A *p*-value > 0.05 was considered indicative of normally distributed data, which are presented as means ± SEM. For group comparisons, independent *t*-tests and one-way ANOVA were conducted. Nonparametric tests, including the Kruskal–Wallis test for multiple comparisons, were applied, with significance set at *p* < 0.05. All experiments were performed in triplicate or more.

## 3. Results

### 3.1. Metabolic Disturbances and Myocardial Injury in Diabetic Rats

**Figure 1:**
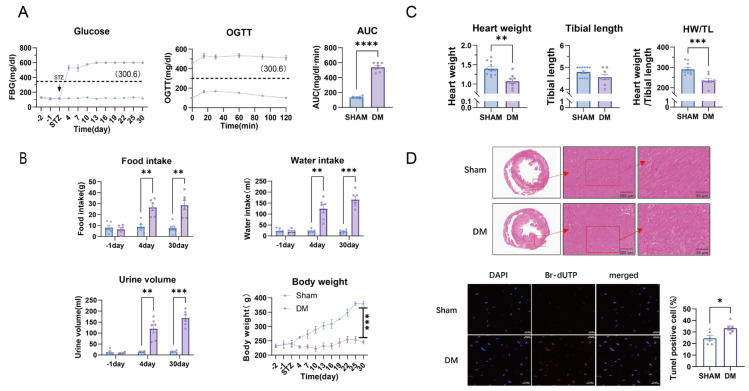
Confirmation of type 1 DM rat model and examination of myocardial structure and apoptosis. (**A**) FBG over 4 weeks (n = 11), OGTT, and mean values of area under the curve of OGTT (n = 8). (**B**) Changes of food intake (FI), water intake (WI), urine volume (UV), and body weight (BW) in sham and DM (n = 6). (**C**) Heart weight (HW) and tibia length (TL) and their ratios in sham and DM. (**D**) H&E staining of rat myocardial tissue (n = 4) and Tunel fluorescence staining results (n = 6). Means ± SEM, * *p* < 0.05, ** *p* < 0.01, *** *p* < 0.001, **** *p* < 0.0001.

After intraperitoneal injection of STZ in SD rats, fasting blood glucose level (FBG, 4 weeks), OGTT and the area under the OGTT curve (AUT) showed significant elevation (Figure 1A), indicating the establishment of DM. Food and water intake as well as excreted urine volume were significantly increased, but body weight was not increased over 4 weeks (Figure 1B), confirming typical phenotypes of type 1 DM. The heart weight (HW) and the ratio of HW to tibia muscle length (TL, HW/TL) were reduced (Figure 1C). Furthermore, hematoxylin and eosin (HE) staining showed interstitial edema in left ventricular (LV) tissue, and TUNEL-positive cells (Br-dUTP in red) were significantly increased in DM (Figure 1D).

### 3.2. Reduced Complex I and V Activities Were Accompanied by Mitochondrial Respiratory Dysfunction in Diabetic Myocardium

**Figure 2:**
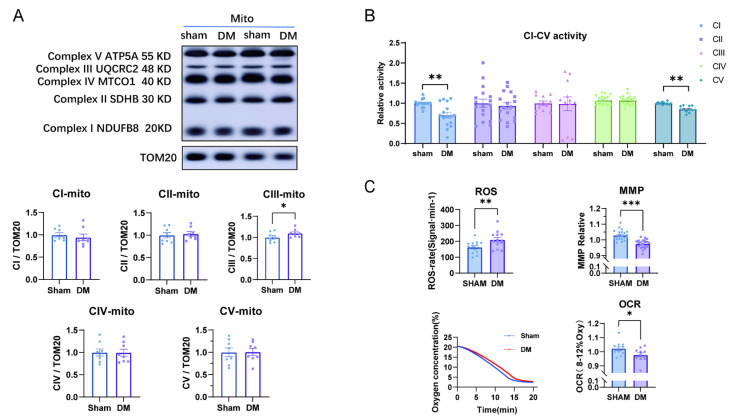
The protein expressions and activities of mitochondrial complexes and mitochondrial function. (**A**) The expressions of mitochondrial complex subunits in sham and DM rat hearts (*n* = 6). Original Western blot images can be found in Figure S2. (**B**) The activity of mitochondrial complexes CI–CV in each group (*n* = 6). (**C**) The level of ROS, MMP in myocardial mitochondria (*n* = 11) and OCR (*n* = 8) in sham and DM. Means ± SEM, * *p* < 0.05, ** *p* < 0.01, *** *p* < 0.001.

Mitochondria were isolated from sham and DM rat hearts. Figure 2A showed that the protein expressions of complexes I, II, IV, and V were unchanged while complex III showed moderate increase in DM. Further evaluation of the activities of cardiac mitochondrial complexes showed significant reduction in that of complex I and complex V in DM (Figure 2B).

Mitochondrial respiration was examined and the results showed that ROS was increased and MMP or OCR were reduced (Figure 2C). These results suggest that there is a tendency of damaged cardiac tissue structure in DM, which is accompanied by reduced mitochondrial respiration and ATP production.

### 3.3. Plasma H_2_S Levels, Cardiac Mitochondrial MPST Protein Expression, and CBS Enzyme Activity Are Decreased in Diabetes

**Figure 3:**
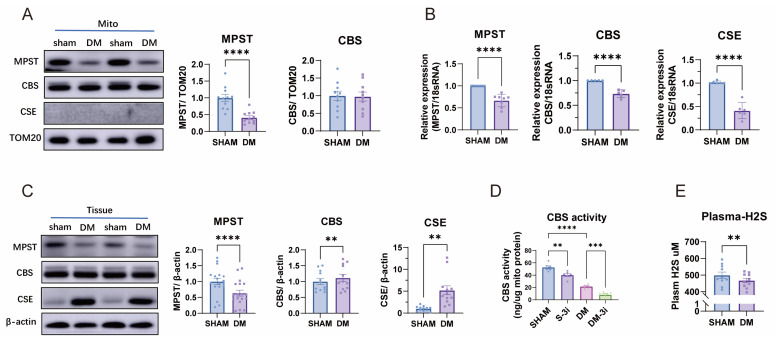
mRNA, protein and activity determination of H_2_S producing enzymes in sham and DM rat heart and mitochondria. (**A**) Protein expressions of MPST and CBS in isolated heart mitochondria of sham and DM (*n* = 6). (**B**) Protein expressions of MPST, CBS, and CSE in sham and DM rat heart tissue (*n* = 6). (**C**) mRNA expressions of CSE, CBS, and MPST in sham and DM rat heart tissue (*n* = 5). (**D**) Activity of CBS in cardiac mitochondria of sham and DM (*n* = 6). (**E**) Plasma H_2_S levels in sham and DM rats (*n* = 8). Original Western blot images can be found in Figure S3. Means ± SEM, ** *p* < 0.01, *** *p* < 0.001, **** *p* < 0.0001.

In isolated mitochondria from the heart tissue, the protein expression of MPST was significantly reduced in diabetic (DM) rats, whereas CBS expression remained unchanged and CSE was undetectable (Figure 3A). A comparable decrease in MPST expression was observed in whole heart tissue from DM rats (Figure 3B). In contrast to mitochondrial findings, both CBS and CSE protein levels were elevated in the hearts of DM rats (Figure 3B). However, at the transcriptional level, the mRNA expression of MPST, CBS, and CSE was consistently downregulated in DM hearts (Figure 3C). Furthermore, mitochondrial CBS activity was markedly reduced in DM rats (Figure 3D), reinforcing the impairment of mitochondrial H_2_S production in diabetes. The purity of mitochondrial extracts was confirmed by the absence of β-actin signal. Consistent with these observations, plasma H_2_S levels were significantly lower in DM rats (Figure 3E), further supporting the notion that systemic H_2_S production is impaired in diabetes [34,35,36]. Collectively, these findings indicate a general dysfunction in H_2_S generation, both at the mitochondrial and systemic levels, in diabetic hearts.

### 3.4. H_2_S Reverses the Decreased Mitochondrial Complex I and V Activity in Diabetic Hearts

**Figure 4:**
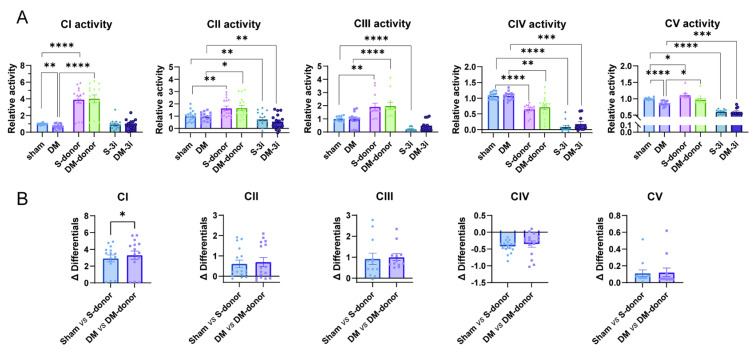
Activities of cardiac mitochondrial complexes I–V and comparisons of the activities of cardiac mitochondrial complexes induced by NaHS. (**A)** The activity of mitochondrial complexes CI–CV under the treatment of H_2_S donor, NaHS, and H_2_S enzyme inhibitors (n = 6). (**B**) Changes in the activity of cardiac mitochondrial oxidative phosphorylation complexes by NaHS in sham and DM (n = 6). Means ± SEM, * *p* < 0.05, ** *p* < 0.01, *** *p* < 0.001, **** *p* < 0.0001.

Reduced H_2_S-producing enzymes or activities imply impaired regulation of mitochondrial functions in DM. Indeed, evaluation of the activity of cardiac mitochondrial complexes showed significant reduction in complex I and complex V in DM at basal level (Figure 4A). We went on and examined H_2_S regulation by using H_2_S donor, NaHS and H_2_S enzyme inhibitors (i3MT, PAG, and HA specifically inhibit MPST, CSE, and CBS, respectively. This combination is herein referred to as “3-inhibitor or 3i”) [37]. As shown in Figure 4A, NaHS (100 µM) significantly increased the activities of complexes I–III and complex V in both sham and DM. Notably, the activity of complex IV was significantly decreased after NaHS (100 µM) treatment (Figure 4A). The activities of complexes I–V were almost diminished by 3-inhibitors (Figure 4A).

Therefore, H_2_S supplementation with NaHS (100 µM) exerted dual effects on mitochondrial complexes: i.e., increased the activities of complexes I, II, III, and V but decreased that of complex IV.

The responses of mitochondrial complexes to NaHS were different, i.e., complex I activity was significantly increased by NaHS in DM rats, whereas other complexes showed similar responses to NaHS in sham and DM (Figure 4B).

### 3.5. H_2_S Enhances Cardiac Mitochondrial Complex Activity by Promoting S-Sulfhydration, with a Greater Effect Observed in Diabetic Rat CardiacMitochondria

**Figure 5:**
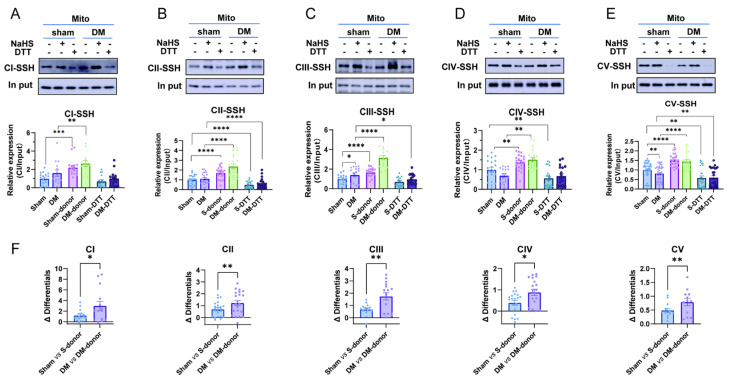
Detection of the *S*-sulfhydration of cardiac mitochondrial complexes I–V and comparisons of the *S*-sulfhydration of cardiac mitochondrial complexes induced by NaHS. (**A**–**E**) The *S*-sulfhydration of cardiac mitochondrial complex I–V under basal conditions and with a H_2_S donor, NaHS (*n* = 6), and an antioxidant, DTT. (**F**) NaHS-induced increases in the *S*-sulfhydration of complexes CI–CV in sham and DM (*n* = 6). Original Western blot images can be found in Figure S4. Means ± SEM, * *p* < 0.05, ** *p* < 0.01, *** *p* < 0.001, **** *p* < 0.0001.

*S*-sulfhydration of complex V is known to mediate H_2_S regulation of its activity. Therefore, we examined whether the observed activities of mitochondrial complexes I–V and their association with H_2_S could be mediated through *S*-sulfhydration. Figure 5A shows the *S*-sulfhydration of mitochondrial complexes I–V under basal conditions. DTT, a reducing agent that reverses *S*-sulfhydration-mediated covalent modification, almost diminished the *S*-sulfhydration of all complexes. Under basal conditions, the *S*-sulfhydration of complex V was reduced but that of complex III was increased in DM heart mitochondria (Figure 5A). NaHS supplementation significantly increased *S*-sulfhydration in all complexes (Figure 5A). These results indicate that the *S*-sulfhydration of mitochondrial complexes can be positively regulated by H_2_S supplementation in sham and DM hearts. Between sham and DM, we observed greater increases in the *S*-sulfhydration of complexes I–V with NaHS in DM (Figure 5B). Since the activities of mitochondrial complexes affected by NaHS were similar (Figure 4B), the data indicate that mitochondrial complexes are vulnerable to H_2_S-mediated post-transcriptional modifications, and supplementation of H_2_S may maintain mitochondrial functions in sham and DM hearts.

### 3.6. H_2_S Enhances Cardiac Mitochondrial Respiration Under Both Basal Conditions and CV Inhibition by Oligomycin

**Figure 6:**
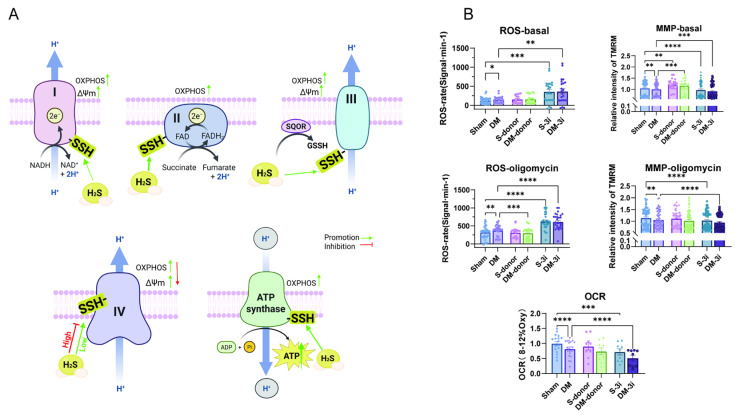
H_2_S regulation of mitochondrial respiration through dual complex responses. (**A**) Schematic diagram presenting H_2_S regulation of the activities of mitochondrial complexes I–V at basal level and with NaHS (100 μM). The activities of complexes I, II, III, and V were all enhanced by NaHS, while the activity of complex IV was decreased at 100 μM. H_2_S-induced complex V activity promotes ATP production. (**B**) ROS, MMP, and OCR levels at basal, with NaHS treatment and 3-inhibitor treatment (*n* = 11). The levels of ROS and MMP response to NaHS or 3-inhibitors after oligomycin treatment (*n* = 11). Means ± SEM, * *p* < 0.05, ** *p* < 0.01, *** *p* < 0.001, **** *p* < 0.0001.

Next, we examined mitochondrial ROS, MMP, and OCR response to H_2_S donor in sham and DM hearts. As shown in Figure 6B, unlike basal conditions, ROS was not increased with NaHS supplementation. MMP, however, remained increased with NaHS in sham and DM. OCR was not decreased with NaHS in DM compared to those of sham. These results suggest that H_2_S supplementation normalizes mitochondrial function between sham and DM.

Complex V inhibition with oligomycin limits ATP synthesis. Figure 6B showed that with oligomycin pre-treatment, ROS level was reduced with NaHS supplementation in DM, and MMP was no longer increased by NaHS (Figure 6B). Therefore, complex V regulation by H_2_S is instrumental in H_2_S regulation of mitochondrial respiration in both sham and DM.

### 3.7. Persulfidation of Cardiac Mitochondrial Complexes Remains Responsive to H_2_S After Ischemia-Reperfusion

**Figure 7:**
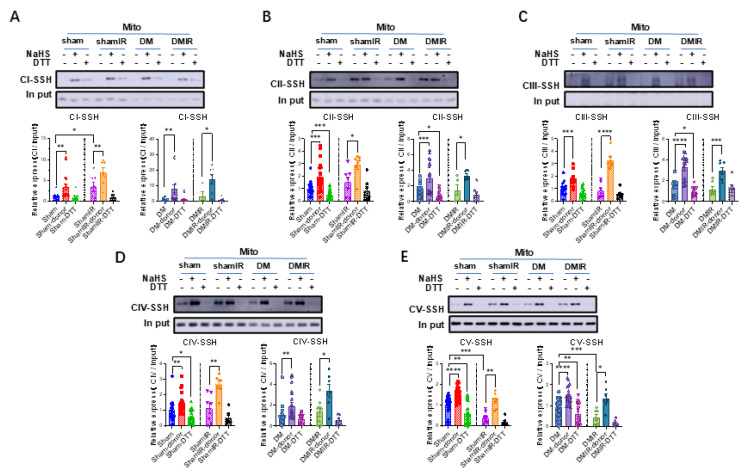
Detection of the S-sulfhydration in cardiac mitochondrial complexes after ischemia-reperfusion. (**A**–**E**) The *S*-sulfhydration levels of cardiac mitochondrial complex CI–CV in sham, sham ischemia-reperfusion (IR), DM, and DM IR (*n* = 5) with NaHS and DTT in sham and DM. Original Western blot images can be found in Figure S5. Means ± SEM, * *p* < 0.05, ** *p* < 0.01, *** *p* < 0.001, **** *p* < 0.0001.

Hyperglycemia and endothelial dysregulation in DM make the heart more susceptible to ischemia-reperfusion (I/R) injury. We went on and examined the impacts of H_2_S regulation of cardiac mitochondrial complexes under the conditions of ischemia-reperfusion in sham and DM. As shown in Figure 7, *S*-sulfhydration of complexes I–V showed similar positive responses to the H_2_S donor, NaHS, in sham and DM with ischemia-reperfusion. *S*-sulfhydration of mitochondrial complexes was inhibited by DTT.

### 3.8. Ischemic-Reperfusion Reduced Complex V Responsiveness to H_2_S in DM Heart and Attenuated Mitochondrial Respiration

**Figure 8:**
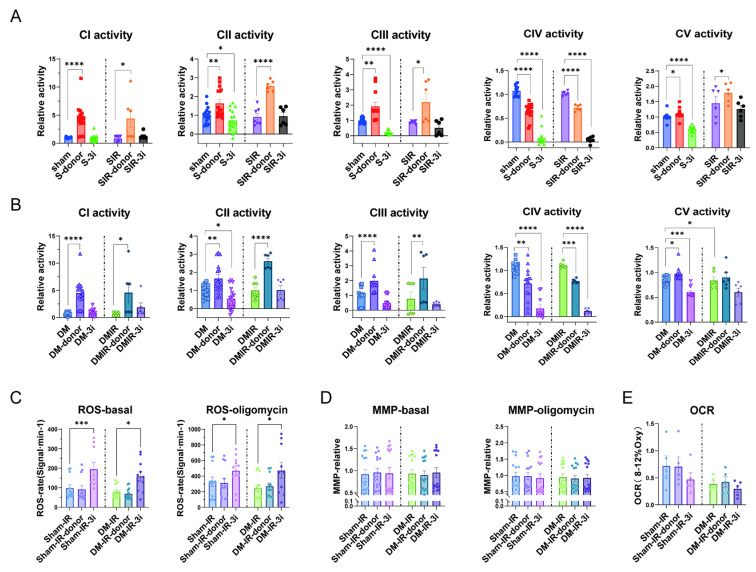
Detection of changes in cardiac mitochondrial complex activity and mitochondrial function after ischemia-reperfusion. (**A**,**B**) The activities of mitochondrial complexes I–V in sham, shamIR, DM, and DMIR (*n* = 6) with NaHS and H_2_S-producing enzyme inhibitors in sham and DM. (**C**,**D**) Determination of mitochondrial ROS and MMP in basal and oligomycin conditions (*n* = 6). (**E**) Determination of mitochondrial OCR in basal condition (n = 4). Means ± SEM, * *p* < 0.05, ** *p* < 0.01, *** *p* < 0.001, **** *p* < 0.0001.

Activities of mitochondrial complexes I–III were increased by NaHS, whereas complex IV activity was inhibited after ischemic-reperfusion (Figure 8A,B). In sham, complex V activity was increased by NaHS after ischemic-reperfusion; however, complex V activity was unaffected in DM (Figure 8B). These results indicate that complex V is vulnerable for ischemia and loses its response to H_2_S after ischemic-reperfusion.

Mitochondrial ROS, MMP, and OCR were examined after ischemic-reperfusion in sham and DM. As shown in Figure 8C–E, the differences between Sham and DM were abolished and NaHS did not alter ROS, MMP, or OCR in either the sham-IR or DM-IR groups. Such responses were maintained with oligomycin pre-treatment. Collectively, these findings demonstrate that ischemic-reperfusion abolished complex V response to H_2_S in DM. However, H_2_S donor failed to affect MMP in both groups, suggesting H_2_S-mediated protective effects may be disrupted following ischemic-reperfusion in the heart.

### 3.9. Conduction Velocity and Diffusion Rate of Electrical Activity Was Impaired After Ischemia-Reperfusion in Sham and DM Hearts

**Figure 9:**
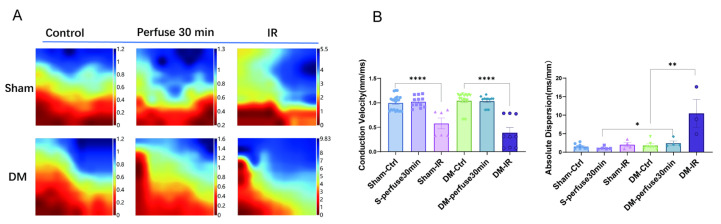
Electrical mapping measurement of electrical conductance and inhomogeneity in sham and DM hearts. (**A**,**B**) Epicardial conduction velocity of monophasic action potentials in sham and DM hearts under normal conditions with continuous perfusion and after ischemia-reperfusion (n = 4). Means ± SEM, * *p* < 0.05, ** *p* < 0.01, **** *p* < 0.0001.

Functional aspects of cardiac function were examined using an electrical mapping system in Langendorff perfused hearts ex vivo. Figure 9A shows graphical image obtained with the mapping system. Averaged electrical conduction velocity in the epicardium of LV was reduced in both sham and DM after ischemia-reperfusion for 30 min. The electrical dispersion (inhomogeneity), which was not different between sham and DM under basal condition, was extravagated in DM in the ischemic-reperfusion group. These results provide evidence of myocardial dysfunction, especially in DM hearts after ischemia-reperfusion.

## 4. Discussion

The current study aims to explore H_2_S regulation of cardiac mitochondrial complexes and the post-translational modification mechanisms in sham and DM rats. Unlike previous studies that focused on single complex functions, our study demonstrates that H_2_S directly regulates mitochondrial complexes I–V and influences mitochondrial bioenergetics. The key findings are as follows: H_2_S-producing enzymes were downregulated in cardiac mitochondria of DM, which was associated with increased mitochondrial ROS and reduced MMP and OCR, indicating impairment of mitochondrial respiration. Under basal conditions, the activities of complex I and V were reduced in cardiac mitochondria of DM but exogenous H_2_S (NaHS) enhanced the activities of mitochondrial complexes I, II, III, and V and reduced complex IV activity in both sham and DM. Using a modified biotin switch assay, we found that *S*-sulfhydrylation of complexes I–IV was not different between sham and DM cardiac mitochondria but *S*-sulfhydration of complex V was significantly reduced in DM under basal conditions. Notably, NaHS increased the *S*-sulfhydration of complexes I–V and NaHS controlled ROS generation and increased MMP. Ischemia-reperfusion did not affect H_2_S regulation of *S*-sulfhydration of complexes I–V or complexes I–IV activities in sham and DM. However, the increase in *S*-sulfhydration caused by H_2_S supplementation after ischemia-reperfusion no longer leads to an increase in complex V activity. ROS measurements revealed that exogenous H_2_S supplementation did not affect ROS generation in either sham-IR or DM-IR groups, whereas inhibition of endogenous H_2_S production significantly increased ROS levels—a trend that persisted in the oligomycin-treated groups. Under basal conditions post-ischemia-reperfusion (IR), neither H_2_S supplementation nor inhibition affected mitochondrial membrane potential (MMP) or oxygen consumption rate (OCR) in sham-IR or DM-IR groups. Similarly, H_2_S showed no effect on MMP under oligomycin treatment. These results indicate that IR causes more severe mitochondrial dysfunction in diabetic conditions, rendering the hearts unresponsive to H_2_S-mediated protection, consistent with the observed alterations in mitochondrial complex activities and protein *S*-sulfhydrylation. Furthermore, IR was found to impair cardiac electrical conduction, particularly in diabetic hearts. In conclusion, this study offers novel insights into the differential effects of H_2_S on mitochondrial function in normal versus diabetic hearts.

Previous studies showed reduced plasma H_2_S levels in STZ-induced DM rats [27,34,35,36], and exogenous H_2_S supplementation can attenuate endothelial dysfunction and oxidative stress [31]. However, evidence has shown that H_2_S production was increased in the pancreas of Zuker diabetic obese rats and STZ-induced diabetic rats, mainly through increased CSE and H_2_S synthesis [38,39]. Consistent with these results, our results also showed that CBS and CSE protein expressions were increased in myocardial tissue of DM but MPST protein expression was reduced. In isolated cardiac mitochondria, MPST protein expression and CBS activity were reduced in DM, while CSE was undetectable, suggesting a downregulation of H_2_S-mediated signaling in the mitochondria of diabetic hearts. Since H_2_S was suggested to be protective of mitochondrial function [40,41,42], it is possible that H_2_S-mediated mitochondrial function was reduced in DM hearts. Indeed, our results showed that mitochondrial respiration was impaired in DM.

Mitochondrial complexes I–V are core components of mitochondrial respiration and biogenesis, and changes in complex activities are prerequisites for mitochondrial dysfunction in diseased conditions. It has been well acknowledged that mitochondrial complexes are actively and reversibly regulated by H_2_S, either with H_2_S as an electron donor transferring electrons to electron transport chain via SQOR or modifying cysteine residues of target proteins through post-translational modification, *S*-sulfhydration. Here, we aimed to comprehensively investigate mitochondrial complexes I–V. Our results clearly showed that the activities of complexes I and V were reduced in DM cardiac mitochondria, but supplementation of H_2_S with its donor, NaHS, significantly in-creased the activities of complexes I, II, III, and V. These results of complex V were consistent with previous results using NaHS as H_2_S donors, which showed enhanced complex activity in the liver of burn injured mice, that is, H_2_S can increase the *S*-sulfhydration level of complex V and thus improve its activity, thereby increasing mitochondrial production capacity [26]. The results collectively suggest that NaHS-enhancement of complex *S*-sulfhydration can promote the activities of complexes I–III and V. The association of H_2_S with mitochondrial complex activities was confirmed with selective inhibitors of H_2_S producing MPST, CBS, and CSE (3-inhibitor) because 3-inhibitor significantly reduced complex activities to minimal level. The effect of H_2_S on complex IV activity, however, is concentration-dependent, i.e., H_2_S at low concentrations (≤50 µM) significantly increased the activity of complex IV but at high concentrations (100 µM–1M) exerted inhibition (Figure S1A). These results are in line with previous reports [22,43] indicating that high concentrations of H_2_S restrict complex IV-mediated electron transport in the mitochondria [43]. Overall, these results indicate that H_2_S may exert dual effects on cardiac mitochondrial complexes: an increase in the activities of complexes I–III and V, but a decrease in the activity of complex IV at high concentrations. Similar dual effects were observed in sham and DM.

Until recently, H_2_S regulation of mitochondrial complexes through *S*-sulfhydration has not been clearly defined. By detecting *S*-sulfhydration of all mitochondria complexes I–V, we found *S*-sulfhydration of mitochondrial complexes I–V in both sham and DM hearts, where complex V was reduced and complex III *S*-sulfhydration was slightly increased in DM under basal conditions. Strong antioxidant DTT abolished the level of *S*-sulfhydration in all complexes, supporting post-translational modifications by H_2_S. In mouse liver mitochondria, *S*-sulfhydration of complex V was associated with greater activity by H_2_S [26]. Indeed, greater *S*-sulfhydration of complexes I–V is associated with increased activities of complexes I, II, III, and V or decreased activity of complex IV. Collectively, post-transcriptional modification, *S*-sulfhydration, leads to changes in the activities of all mitochondrial complexes in normal and DM hearts.

Compared to sham, significantly higher levels of *S*-sulfhydration were observed with NaHS in mitochondrial complexes in DM. The activities, however, were not different between sham and DM. The effects were observed under conditions in which the mitochondrial complex amount remained unchanged in two groups. Therefore, H_2_S regulated the activities of cardiac mitochondrial complexes in DM by significantly increasing *S*-sulfhydration of complex proteins. Nevertheless, in the presence of NaHS, cardiac mitochondrial ROS was no longer different between sham and DM, but MMP was increased in two groups, confirming the enhancement of mitochondrial function. H_2_S donors are shown to increase MMP when targeted to mitochondria [44,45], which is consistent with our results. Inhibition of complex V with oligomycin abolished H_2_S increment of MMP, while ROS was unchanged, indicating that complex V regulation by H_2_S (i.e., biogenesis) is an important process maintaining the overall mitochondrial activity. It is noted that NaHS inhibition of complex IV is instrumental in moderating overall mitochondrial respiration because it may control electron transport and ROS generation secondary to increased complexes I–III activities.

H_2_S regulation of mitochondrial complexes was observed under the conditions of ischemia reperfusion due to the vulnerability to ischemic-reperfusion injury of DM hearts secondary to hyperglycemia and endothelial dysfunction in coronary circulation. Responses of mitochondrial complexes to H_2_S were examined following 30 min non-perfusion followed by 30 min reperfusion in both sham and DM. First of all, *S*-sulfhydration of complex V was reduced following ischemia-reperfusion in sham and DM and NaHS was able to increase the *S*-sulfhydration of mitochondrial complexes in both groups. These results suggest that at least in type 1 DM, H_2_S-regulated post-transcriptional modification is maintained in mitochondrial complexes. The responses to NaHS of mitochondrial complexes I–IV were largely unaffected after ischemia-reperfusion in both sham and DM. However, complex V activity was unable to increase with NaHS supplementation. Therefore, complex V is vulnerable to ischemia and may be responsible for reduced mitochondrial ATP production and subsequent cardiac damage.

H&E examination results showed interstitial edema in DM LV myocardium with increased TUNEL staining, suggesting cardiomyocyte apoptosis. While we did not directly assess mitophagy or mitochondrial dynamics in DM hearts, elevated PINK1 and BNIP3L protein levels accompanied by reduced OPA1 (Figure S1B,C) suggest ROS-triggered mitophagy activation and cardiac injury [46]. Regarding mitochondrial dynamics, MFN2 upregulation without MFN1 alteration implies a potential shift toward mitochondrial fusion. The unchanged expression of fission regulators DRP1 and FIS1 further supports the idea that diabetic cardiomyocytes may promote mitochondrial fusion as an adaptive response to metabolic stress (Figure S1B,C). Functional assays were conducted and confirmed that myocardial conduction velocity, especially absolute dispersion inhomogeneity of the heart, was affected in DM. Diabetes can lead to severe cardiomyocyte metabolic disorders, cardiac hypertrophy, microcirculatory dysfunction, and increased oxidative stress [47]. Echocardiography and magnetic resonance spectroscopy studies have shown that even in diabetic patients with normal cardiac function, the creatine phosphate/ATP ratio is reduced [48], suggesting that mitochondrial dysfunction may precede affected myocardial contractile function [46].

It should be noted that *S*-sulfhydration of mitochondrial complex proteins by H_2_S donors was maintained in the hearts after ischemia-reperfusion. This new insight into the role of H_2_S in mitochondrial bioenergetics provides a promising direction for the development of new therapies to improve mitochondrial function in diabetic cardiomyopathy.

## 5. Conclusions

H_2_S is an important regulator of mitochondrial function in both normal and DM hearts. We provided direct evidence to show that *S*-sulfhydration of complexes are as-sociated with H_2_S regulation of complex activities. Furthermore, H_2_S exerted dual effects on mitochondrial respiration, predominantly enhancing electron transport and ATP synthesis via complexes I–III and complex V but restricting electron transport through complex IV. The coordination of mitochondrial complexes is important in maintaining mitochondrial respiration and therefore protection from abnormal electron flux and oxidative stress. Our study provides new insights into the potential of H_2_S as a therapeutic agent to alleviate mitochondrial dysfunction and cardiac protection in type 1 DM.

## Data Availability

The original contributions presented in this study are included in the article/Supplementary Materials. Further inquiries can be directed to the corresponding author.

## Supplementary Materials

The following supporting information can be downloaded at: https://www.mdpi.com/article/10.3390/biom15081197/s1, Figure S1: (a) CIV activity (b) Mitophagy (c) Mitochondria Fission & Fusion; Figures S1–S5: Original Western blot images.

## Abbreviations

The following abbreviations are used in this manuscript:

Abbreviation	Full Name
T1DM	Type 1 diabetes mellitus
H_2_S	Hydrogen sulfide
STZ	Streptozotocin
BW	Body weight
FI	Food intake
WI	Water intake
UV	Urine volume
FBG	Fasting blood glucose
OGTT	Oral glucose tolerance test
OCR	Oxygen consumption rate
ROS	Reactive oxygen species
MMP	Mitochondrial membrane potential
HWI	Heart mass index
TL	Tibial length
MPST	Mercaptopyruvate thiotransferase
CBS	Cystathionine-beta-synthase
CSE	Cystathionine-gamma-lyase
NaHS	Sodium hydrosulfide
DTT	Dithiothreitol
HA	Hydroxylamine
PAG	DL-Propargylglycine
MMTS	S-Methyl methanethiosulfonate
DDW	Double-distilled water
HE	Hematoxylin-eosin staining
ELISA	Enzyme-linked immunosorbent assay

## Appendix A

**Table A1:** List of antibodies used in the study.

Protein Target	Manufacturer	Catalogue Number	Concentration Used
OXPHOS	Abcam (Cambridge, UK)	Ab110413	6.0 μg/mL
PINK1	Novus (Chesterfield, MI, USA)	BC100-494	1:2000
BNIP3L	Thermo Fisher (Pittsburgh, PA, USA)	PA5-114914	1:1000
DRP1	Cell signaling (Boston, MA, USA)	8570S	1:1000
FIS1	Thermo Fisher (Pittsburgh, PA, USA)	Ab42364	1:1000
OPA1	Abcam (Cambridge, UK)	Ab42364	1:1000
MFN1	Thermo Fisher (Pittsburgh, PA, USA)	PA5-38042	1:1000
MFN2	Abcam (Cambridge, UK)	Ab124773	1:1000
β-actin	Santa Cruz (Paso Robles, CA, USA)	Sc-47778	1:1000
TOMM20	Santa Cruz (Paso Robles, CA, USA)	Sc-17764	1:1000
MPST	Santa Cruz (Paso Robles, CA, USA)	Sc-376168	1:1000
CBS	Santa Cruz (Paso Robles, CA, USA)	Sc-133154	1:1000
CSE	Santa Cruz (Paso Robles, CA, USA)	Sc-365381	1:1000

**Table A2:** Primer list.

Primer Name	Primer Sequence	Primer Length
18s RNA-F	CGCGGTTCTATTTTGTTGGT	20
18s RNA-R	AGTCGGCATCGTTTATGGTC	20
MPST-F	CCGAGCTCTGGTATCTGCTC	20
MPST-R	ACCAGGACGCATCCAGTAAC	20
CSE-F	CTCTGGAAATCCGACGAGGA	20
CSE-R	AGGCGAAGGTCAAACAGTGC	20
CBS-F	CAATACCGCAACAATGGCGT	20
CBS-R	TGCCACGAAGTTTAGCAGGT	20
