# Supplementary material for: Dual Regulation of Mitochondrial Complexes by H2S via S-Sulfhydration Controls Respiration in Type 1 Diabetic Hearts
Source: Biomolecules. 2025 Aug 20;15(8):1197. doi: 10.3390/biom15081197 (PMC12384692; doi:10.3390/biom15081197)
Supplement: Supplementary file 1 [file biomolecules-15-01197-s001.zip › biomolecules-3706119 WB original images.pptx]

## Slide 1
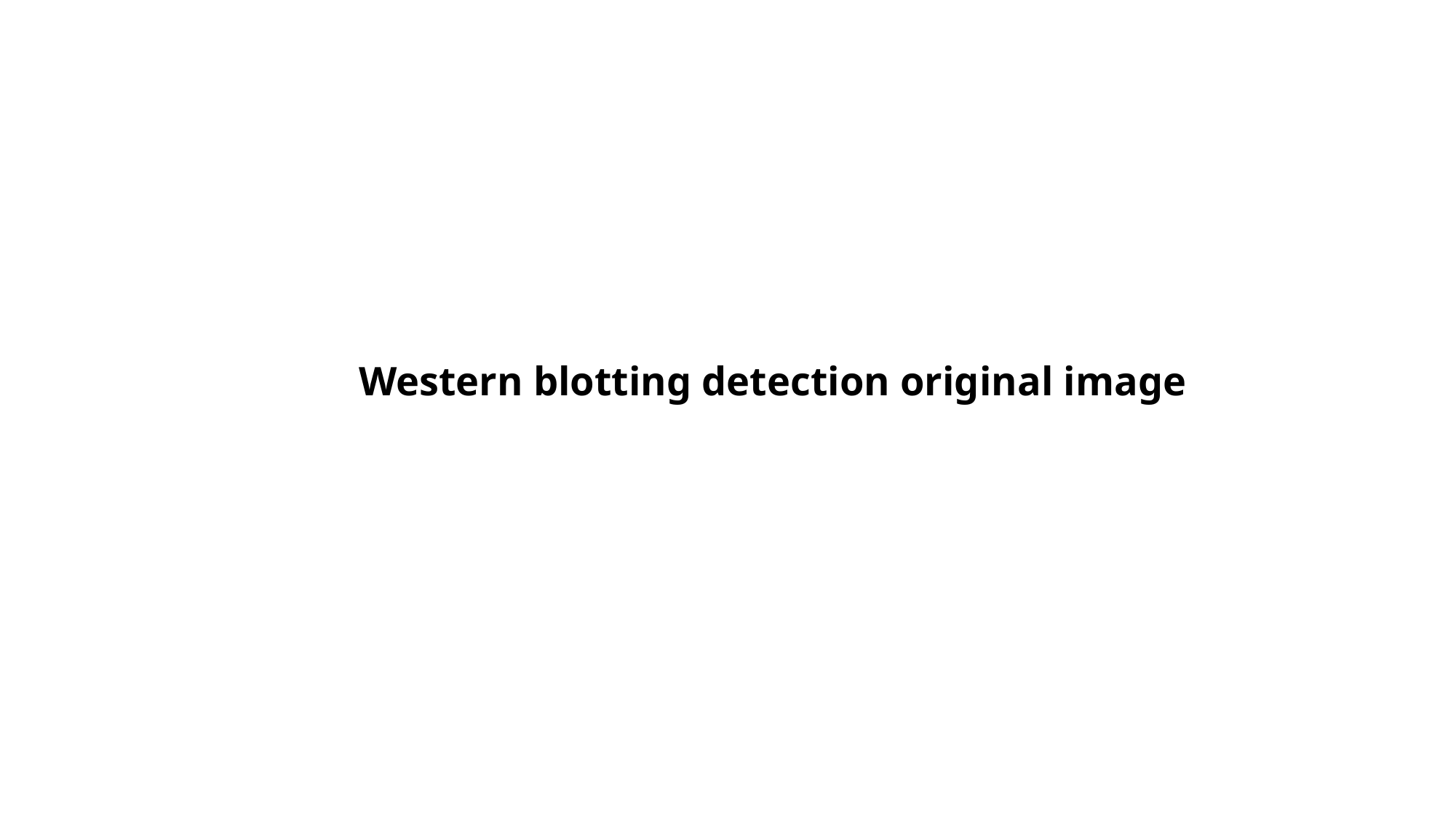

Western blotting detection original image

## Slide 2
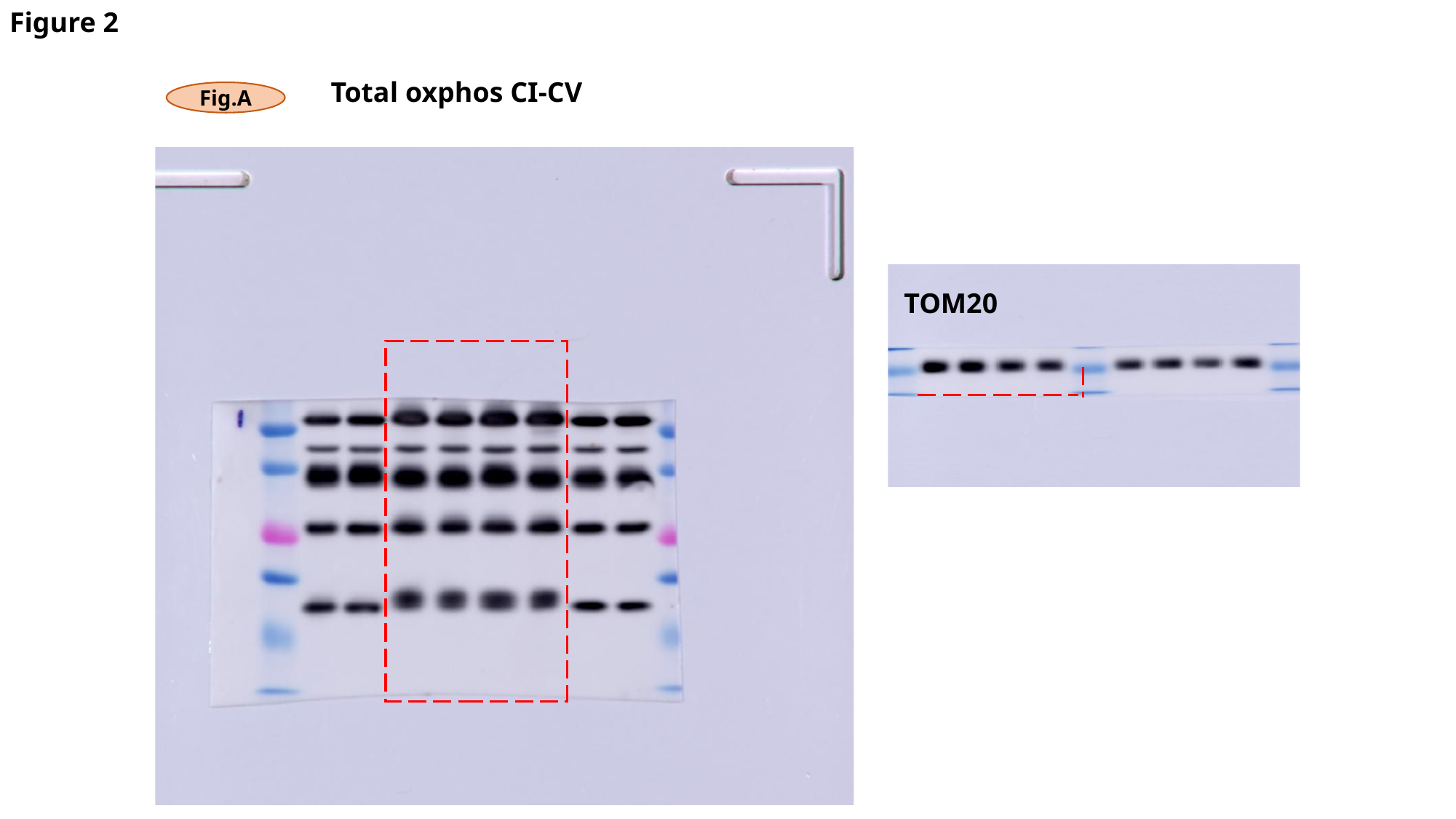

Figure 2
Total oxphos CI-CV
Fig.A
TOM20

## Slide 3
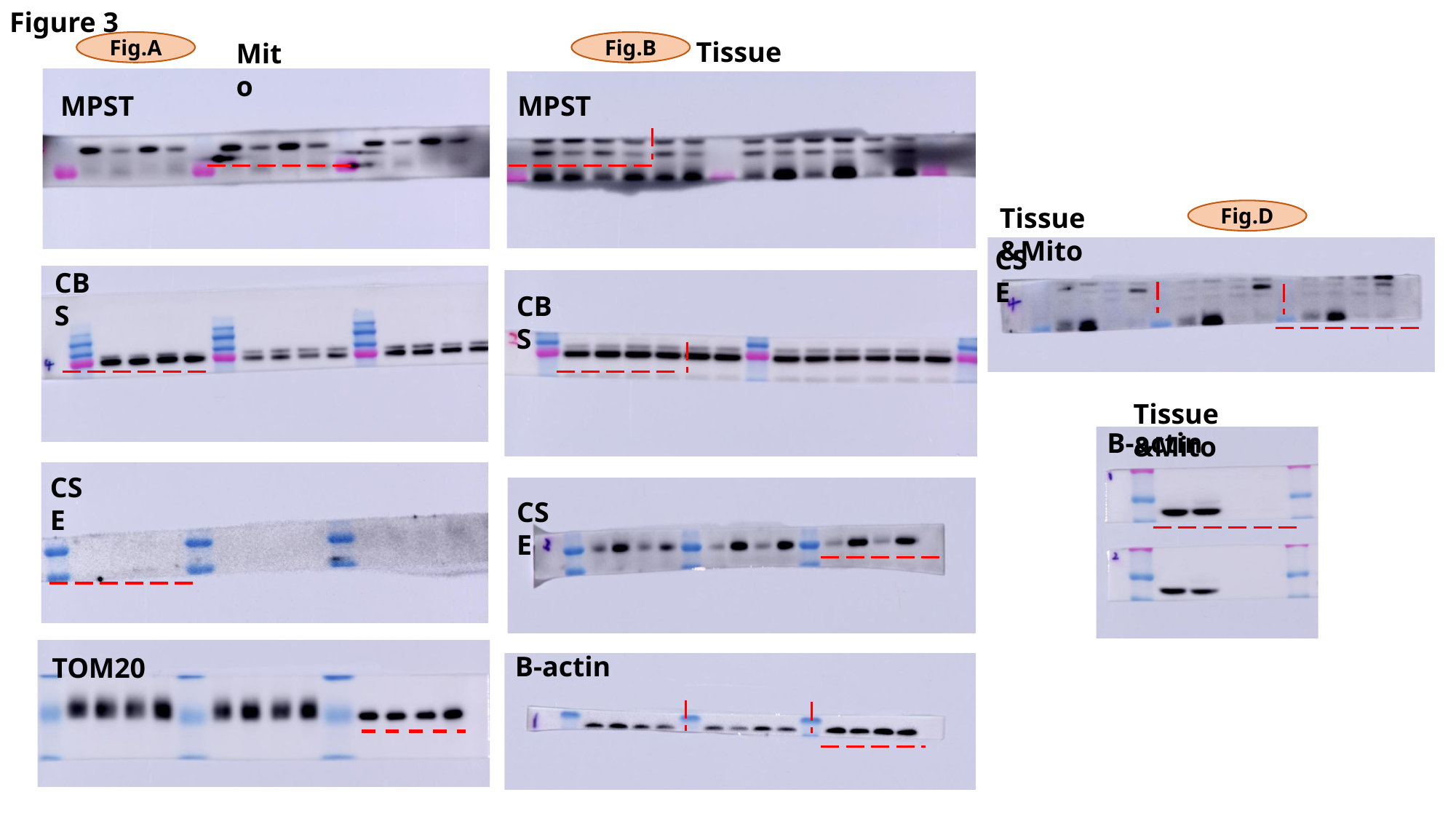

Figure 3
Tissue
Mito
Fig.A
Fig.B
MPST
MPST
Tissue &Mito
Fig.D
CSE
CBS
CBS
Tissue &Mito
B-actin
CSE
CSE
B-actin
TOM20

## Slide 4
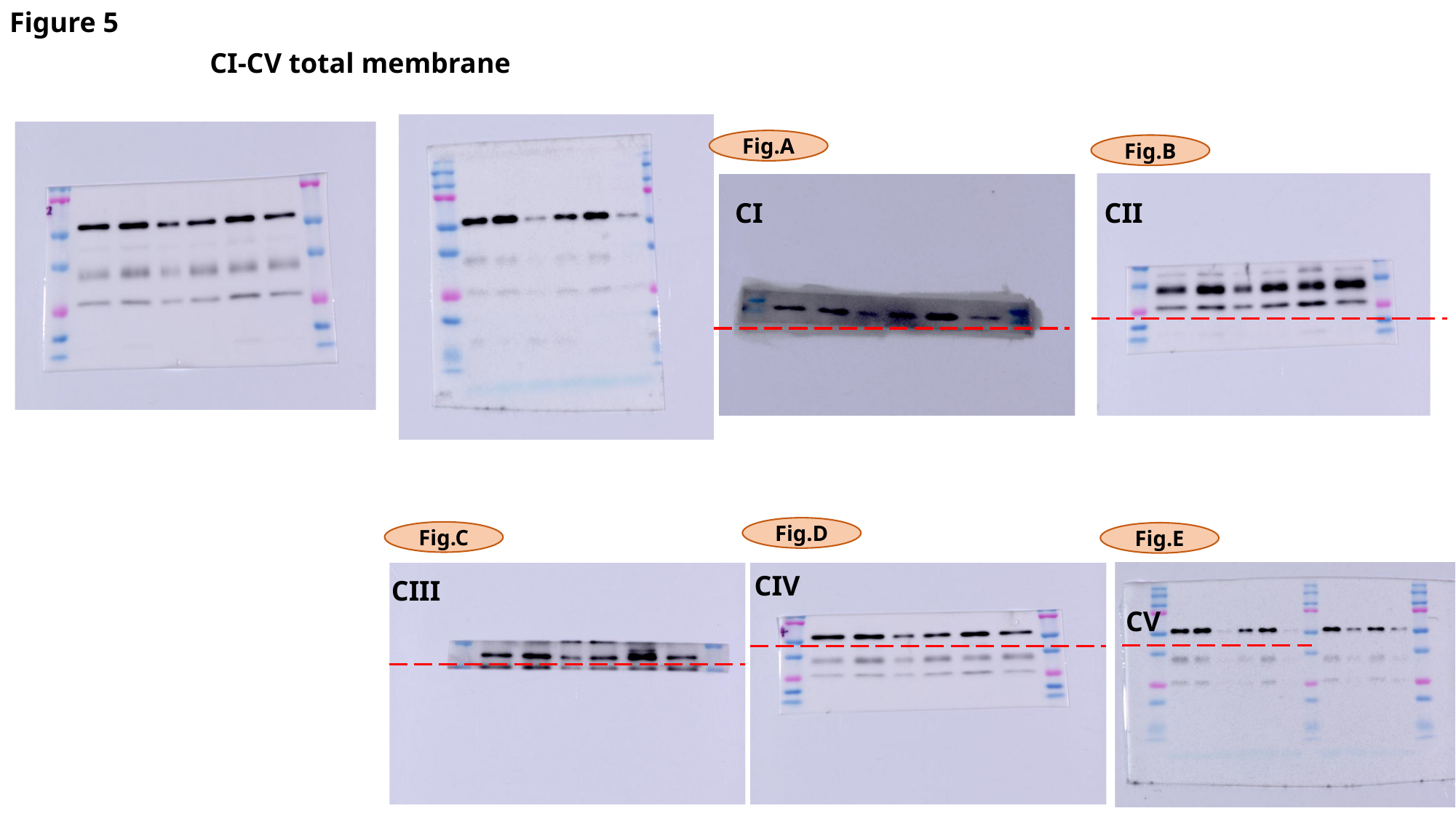

Figure 5
CI-CV total membrane
Fig.A
Fig.B
CI
CII
Fig.D
Fig.C
Fig.E
CIV
CIII
CV

## Slide 5
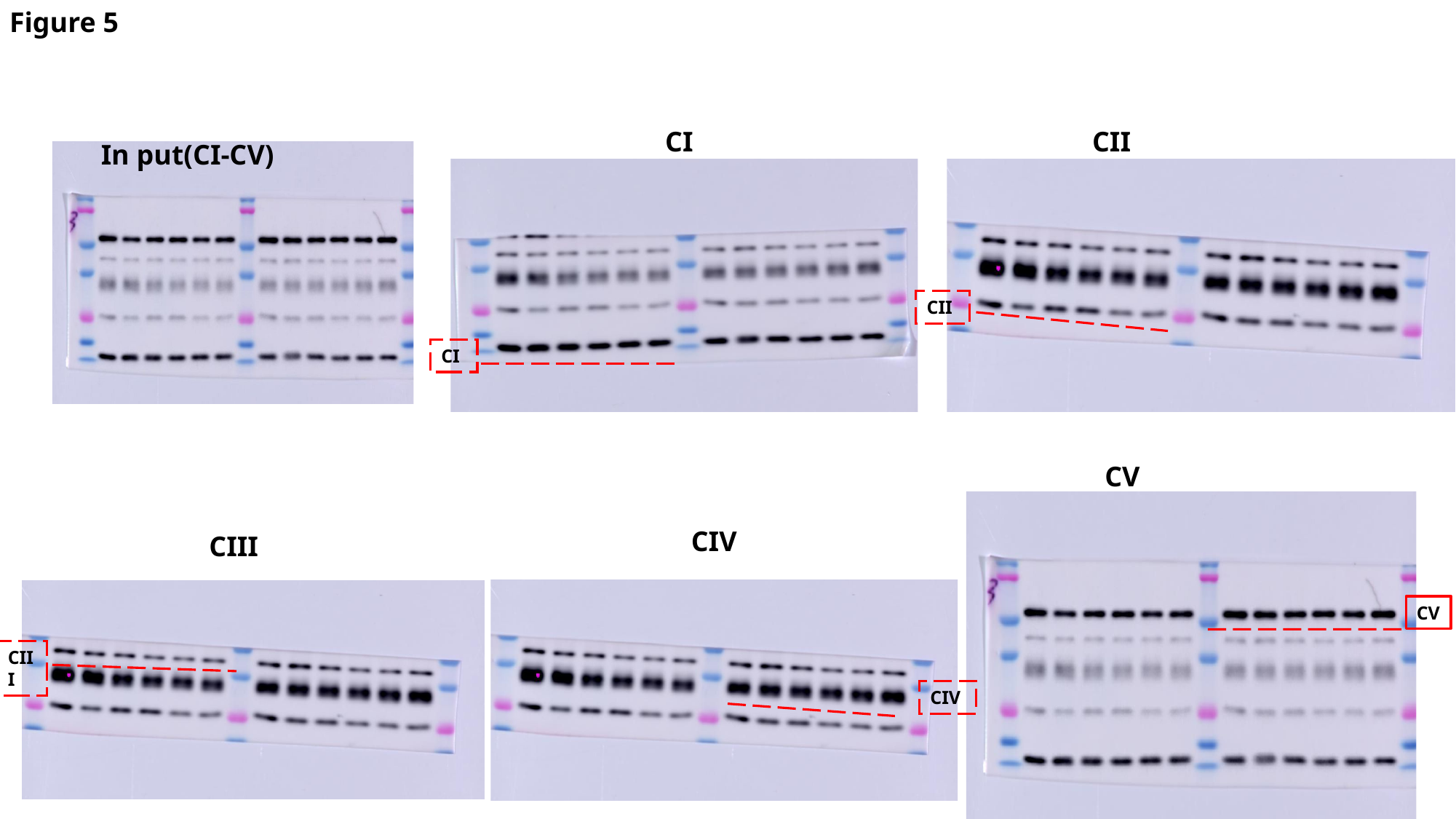

Figure 5
CII
CI
In put(CI-CV)
CII
CI
CV
CIV
CIII
CV
CIII
CIV

## Slide 6
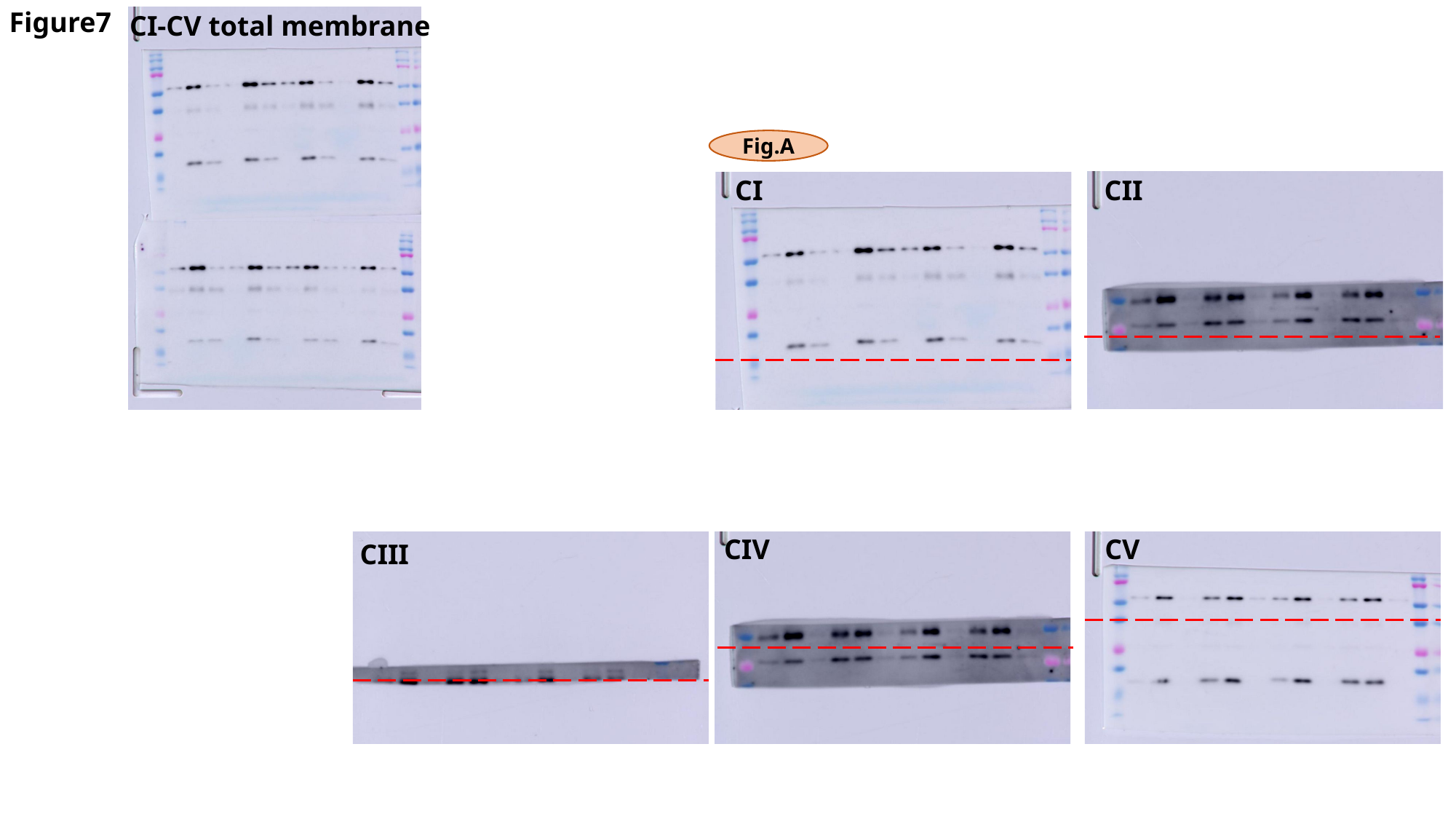

Figure7
CI-CV total membrane
Fig.A
CI
CII
CV
CIV
CIII

## Slide 7
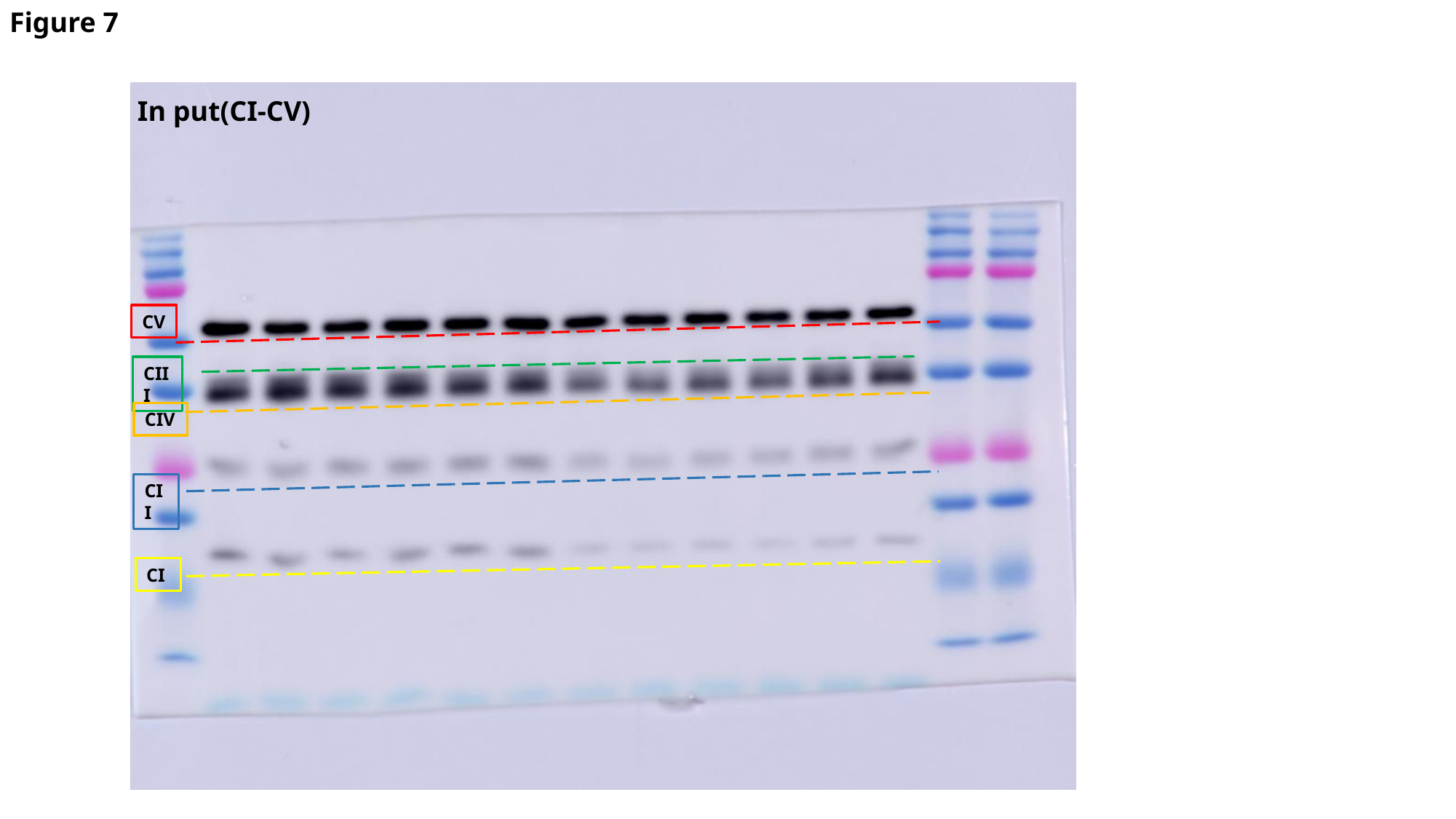

Figure 7
In put(CI-CV)
CV
CIII
CIV
CII
CI

## Slide 8
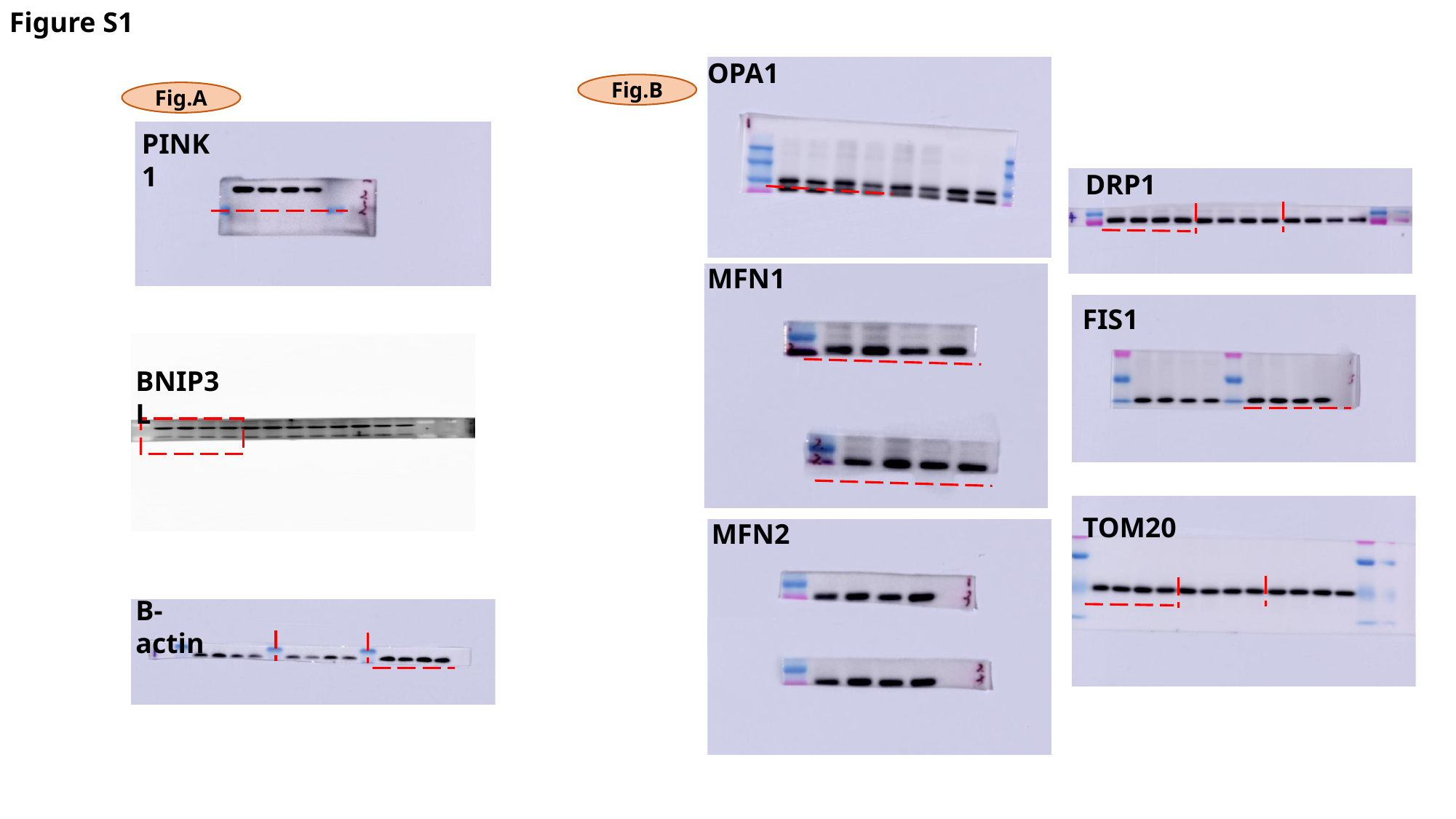

Figure S1
OPA1
Fig.B
Fig.A
PINK1
DRP1
MFN1
FIS1
BNIP3L
TOM20
MFN2
B-actin
