# Supplementary figures and images for: Dual Regulation of Mitochondrial Complexes by H2S via S-Sulfhydration Controls Respiration in Type 1 Diabetic Hearts
Source: Biomolecules. 2025 Aug 20;15(8):1197. doi: 10.3390/biom15081197 (PMC12384692; doi:10.3390/biom15081197)

# A

## CIV activity

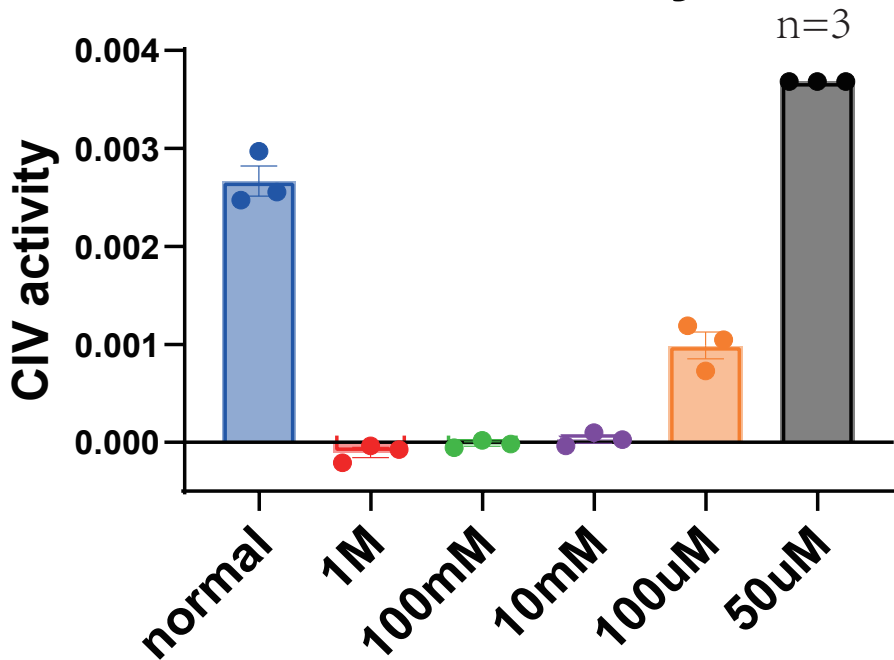

Supplement: Supplementary file 1 [file biomolecules-15-01197-s001.zip › Figure S1A - CIV activity.pdf]

# B

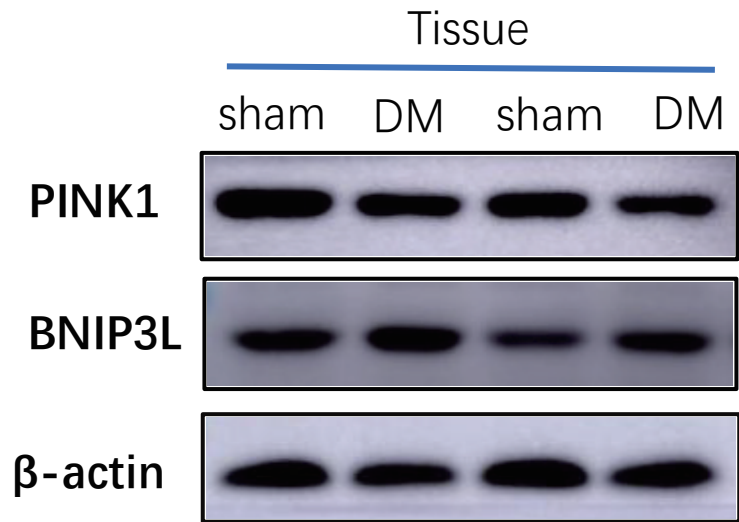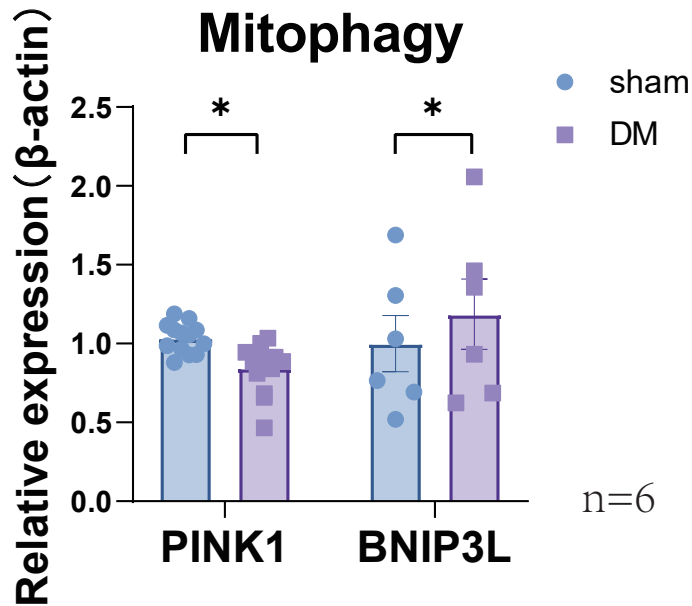

Supplement: Supplementary file 1 [file biomolecules-15-01197-s001.zip › Figure S1B - Mitophagy.pdf]

C

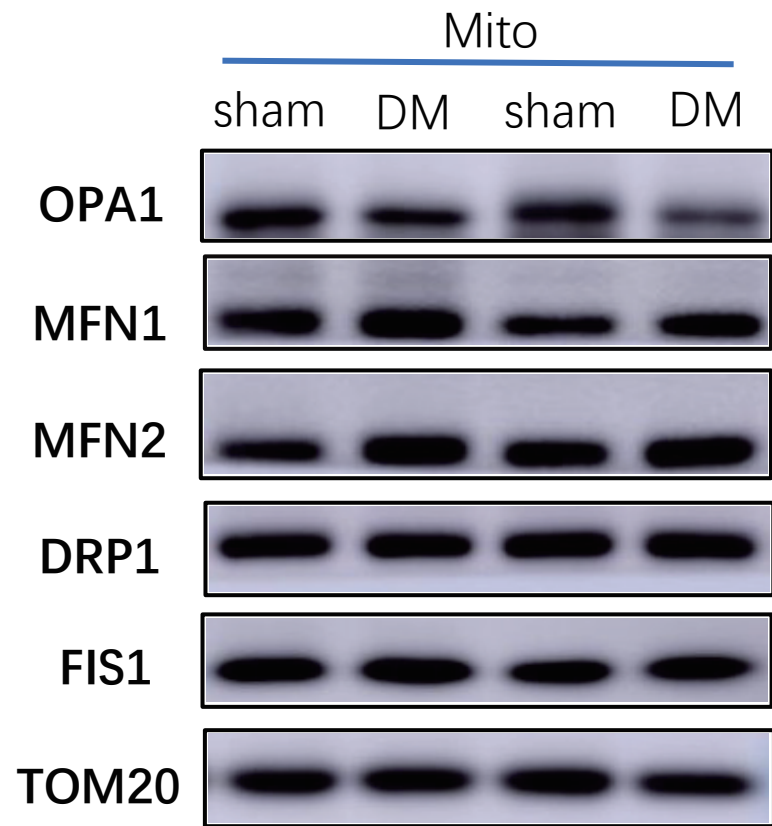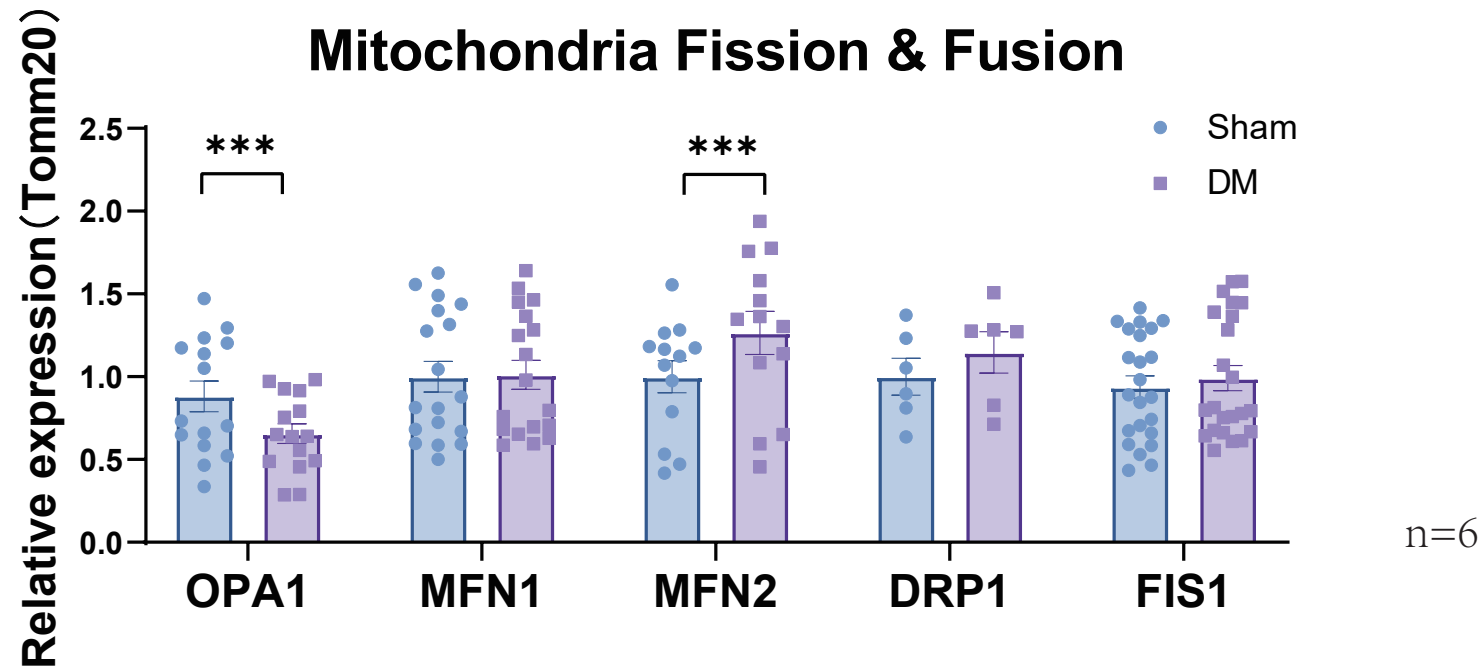

Supplement: Supplementary file 1 [file biomolecules-15-01197-s001.zip › Figure S1C - Mitochondria Fission & Fusion.pdf]
